# Homœopathic Medical College of Missouri

**Published:** 1893-01

**Authors:** 


### HOMŒOPATHIC MEDICAL COLLEGE OF MISSOURI.

The regular winter session, 1892–93, of this institution was opened on September 22d, with a short address by the Dean, Dr. William C. Richardson, which was followed by an interesting and instructive opening lecture delivered by Prof. W. L. Reed, M. D.

Prof. Richardson in his remarks called attention to the determination of the faculty to continue the same kind of thorough homœopathic instruction which has gained for the old college such enviable reputation, and placed her graduates in the foremost ranks of learning throughout the civilized world.

He further announced that he was authorized by an honored graduate, Dr. William D. Gentry, of Chicago, to offer as a prize a full set of the *Concordance Repertory of the Materia Medica*, bound in half Russia, worth forty-two dollars, as a Materia Medica prize to the graduate each year, who shall pass the best examination in Materia Medica on a physiological basis.

Dr. Gentry does this because of his affectionate regard for his *Alma Mater*, and a wish to stimulate students in the essentials of Homœopathy.

Prof. Reed's lecture was replete with statistics, showing the advantages of the homœopathic practice, and he particularly called attention to the undisputed and undeniable superiority of Homœopathy in the treatment of cholera.

His effort elicted profound attention and called forth hearty applause.

The class for this session is the largest that has been enrolled for several years, and the prospects are exceedingly good.

